# Temporal dynamics of rumination in daily life: a multi-method investigation of attentional control and metacognitive pathways toward depression

**DOI:** 10.3389/fpsyt.2026.1900459

**Published:** 2026-08-17

**Authors:** Yuanyuan Zeng, Zini Chen, Qingzi Zhu, Lijing Niu, Xiayan Chen, Haowei Dai, Bihua Zhou, Xiaoling Peng, Ruibin Zhang

**Affiliations:** 1 Laboratory of Cognitive Control and Brain Healthy, Department of Psychology, School of Public Health, Southern Medical University, Guangzhou, China; 2 School of Laboratory Medicine and Biotechnology, Southern Medical University, Guangzhou, China; 3 College of Teacher Education, East China Normal University, Shanghai, China; 4 Guangzhou Cana School, Guangzhou Rehabilitation and Research Center for Children With ASD, Guangzhou, China

**Keywords:** attentional control, depressed mood, Ecological Momentary Assessment, positive metacognitive beliefs, rumination

## Abstract

**Background:**

Rumination is a central mechanism in depression maintenance. A substantial body of research indicates that deficits in attentional control and positive metacognitive beliefs jointly contribute to this depressive rumination. However, these studies have been largely confined to cross-sectional and between-person designs, limiting their ability to capture the dynamic, within-person fluctuations of these relationships and leaving the within-person pathways through which they influence rumination and mood poorly understood.

**Methods:**

We employed a multi-method design. Study 1 used cross-sectional path analysis (N = 329) to test rumination as a mediator between attentional control (focusing/shifting), positive metacognitive beliefs, and depressive symptoms. Study 2 implemented a 14-day Ecological Momentary Assessment (EMA; N = 135, 70 timepoints/person) analyzed with Dynamic Structural Equation Modeling (DSEM) to examine contemporaneous (*t*) and lagged (*t*-1) effects.

**Results:**

Cross-sectional results from Study 1 revealed rumination served as a mediator in the relationships between both attentional control (focusing/shifting) and positive metacognitive beliefs with depressive symptoms. DSEM in Study 2 revealed a temporal cascade: positive metacognitive beliefs and impairments of attentional control (focusing/shifting) predicted the increase of rumination at same time-point; rumination, in turn, further predicted an increase in negative affect both concurrently and at the subsequent time-point.

**Conclusions:**

These findings suggest that rumination serves as within-person dynamic pathways through which attentional control deficits and positive metacognitive beliefs predict elevated depressed mood. This temporally ordered process provides evidence for the cognitive mechanisms underlying depressed mood fluctuations and may inform future research on time-sensitive intervention frameworks.

## 1 Introduction

As a highly prevalent and disabling mental disorder, depression poses a serious challenge to global public health. According to the Global Burden of Disease Study in 2021, approximately 229 million people worldwide suffered from major depressive disorder. It ranks among the most burdensome mental disorders and is the second leading cause of disability globally (1). Despite advances in pharmacological and psychological treatments, relapse rates remain high and many individuals experience persistent symptoms (2), highlighting the urgent need to better understand the mechanisms underlying the maintenance and recurrence of depression. A growing consensus points to emotion dysregulation, marked by persistent negative affect and reduced positive affect, as a fundamental driver of depressive chronicity. This dysregulation is often fueled and maintained by maladaptive cognitive processes, most prominently rumination, which amplifies negative affect, impairs positive engagement, and disrupts effective emotion regulation (3).

According to the Response Styles Theory (4), individuals characterized by a ruminative response style tend to react to their own negative mood states by passively and repeatedly dwelling on them, a process termed depressive rumination (5). As a maladaptive emotion-regulation strategy (6), rumination can, over time, evolve into a habitual response to negative affect, contributing to the emergence and maintenance of depression (7). Consistently, rumination can predict the onset (8, 9), severity (10, 11) and reoccurrence (12) of depression. Moreover, neuroimaging evidence confirmed that rumination and depression share a specific neural basis, with rumination consistently activating core regions of the brain’s default mode network (13). Importantly, interventions that target rumination, such as metacognitive therapy, were proven effective in alleviating depression (14). Herein, clarifying what triggers rumination in daily life and why it persists from one moment to the next is therefore critical for theory and intervention of depression.

The *Impaired Disengagement Hypothesis* (IDH) proposed impaired conflict signaling and impaired attentional control to account for heightened levels of rumination observed in depression (15). The IDH conceptualized attentional control broadly, with its inhibitory function being central to the proposed deficits. Insufficient inhibition is thought to contribute to sustained, passive occupation of working memory by negative self-referential content, which facilitates repetitive, maladaptive brooding. This proposed cognitive mechanism may link deficient top-down control to the persistence of ruminative thought—a process further elucidated by Koster et al. (16). Neuroimaging evidence indicates that rumination and inhibitory control are linked through shared neural substrates, an association explicable by both overlapping brain networks and the antagonistic relationship between the default mode and frontoparietal networks (17). As research suggests attentional control primarily consists of two facets (i.e., focusing and shifting; 18). Attentional focusing refers to the sustained engagement with a task, and attentional shifting refers to the flexible reallocation of focus between tasks (19). Deficits in attentional shifting (19–21) and focusing (22, 23) have been identified as predictors of rumination. Salguero et al. (24) empirically validated and extended the IDH by examining attentional control and positive metacognitive beliefs as key components. The metacognitive model of psychopathology (the Self-Regulatory Executive Function model, S-REF; 25)) posits that emotional distress is maintained by a set of maladaptive strategies termed the Cognitive Attentional Syndrome (CAS), which includes perseverative thinking (e.g., rumination), threat monitoring, and dysfunctional coping behaviors. The CAS is directed by metacognitive beliefs, i.e., individuals’ beliefs about their own thinking. Positive metacognitive beliefs reflect the perceived utility of rumination (e.g., “ruminating helps me solve my problems”), whereas negative metacognitive beliefs concern the uncontrollability and danger of one’s thoughts. Within the S-REF framework, positive metacognitive beliefs are thought to activate and sustain the CAS by motivating individuals to engage in rumination as a coping strategy, thereby paradoxically maintaining emotional distress (25, 26). Furthermore, empirical studies have also indicated that rumination mediates the association between positive metacognitive beliefs and depression (27, 28).

A substantial body of research has confirmed that both attentional control and positive metacognitive beliefs explain elevated rumination in depression. However, these studies are primarily cross-sectional in design, which precludes the tracking of dynamic changes in these relationships over time. Furthermore, being limited to between-person analyses, they cannot capture within-person fluctuations. Such within-person dynamics not only transcend simple daily associations but also embody the unique, core mechanisms at the individual level that explain psychopathological processes (29). In recent years, some studies have begun to employ Ecological Momentary Assessment (EMA) to examine temporal associations. For instance, Yaroslavsky et al. (30) found that attentional control deficits predicted higher levels of daily negative affect, however, the attentional deficits in this study were conceptualized and analyzed as a between-person (trait-like) variable. Similarly, Tamm et al. (31) used EMA finding the fluctuations in rumination were directly predicted by prior ruminative state, attentional control and positive metacognitive beliefs, yet did not analyze the mediating role of rumination between these predictors and depressed mood. In addition, Cano-López et al. (32) has confirmed the day-to-day and within-day mediating role of metacognitive strategies between positive metacognitive beliefs and negative affect at the within-person level, using Dynamic Structural Equation Modeling (DSEM). However, the day-level temporal resolution may be insufficient to capture shorter-term fluctuations. Consequently, no study to date has examined, in daily life at the within-person level, the three distinct dynamic pathways by which attentional focusing, attentional shifting and positive metacognitive beliefs relate to depressed mood through rumination in daily life.

**Figure 1:**
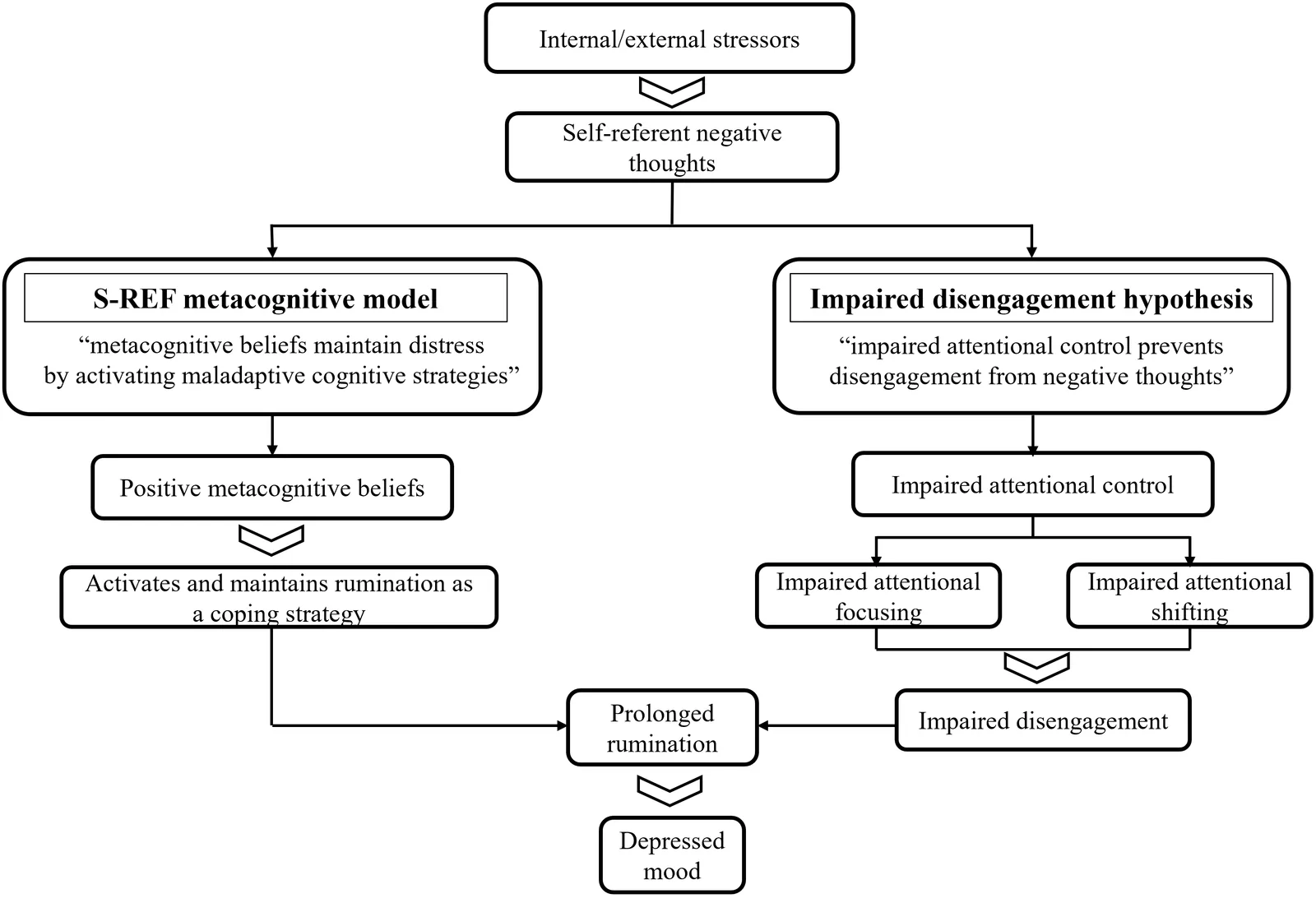
Conceptual model of cognitive pathways to rumination and depressed mood.

To advance knowledge in this domain, the present research employs a multi-method design. Study 1 adopted a cross-sectional design to validate the extension of the IDH proposed by Salguero et al. (24) and to systematically examine the stable, between-person relationships among attentional control, positive metacognitive beliefs, rumination, and depression. Building on this static foundation, Study 2 implemented a 14-day EMA protocol (five times daily at three-hour intervals) combined with DSEM, aiming to examine the dynamic mediation processes of the three cognitive pathways (see **Figure 1**) at the within-person level. Specifically, separate DSEM analyses were conducted for the three hypothesized pathways: from attentional focusing to depressed mood via rumination; from attentional shifting to depressed mood via rumination; from positive metacognitive beliefs to depressed mood via rumination. Our models simultaneously estimated both contemporaneous and lagged effects while controlling for each variable’s prior state, thereby accounting for temporal inertia. This approach first allows us to trace the within-person, time-varying fluctuations of these three cognitive-affective pathways in daily life. Second, by leveraging the person-mean centering and random effects inherent to DSEM, it enables us to disentangle within-person processes from between-person differences, thereby isolating dynamic mechanisms operating at the individual level. Finally, the method also supports the generation of time-lagged evidence consistent with temporally ordered associations.

## 2 Study 1

Study 1 aimed to examine the stable, between-person mediating role of rumination in the relationships among attentional control, positive metacognitive beliefs, and depression, employing a cross-sectional design.

### 2.1 Method

#### 2.1.1 Participants and measures

Study 1 comprised 329 undergraduate students recruited from Southern Medical University through campus advertisements and online bulletins (216 females, 65.7%; 113 males, 34.3%; age range: 17–30 years, M = 21.49, SD = 1.53). After providing electronic informed consent via a secure platform, participants completed cross-sectional assessments including the Chinese version of the Attentional Control Scale (33), Positive Beliefs About Rumination Scale (34), Ruminative Responses Scale (4), and Patient Health Questionnaire-9 (PHQ-9) (35). All the scales were validated with acceptable reliability and validity in the Chinese context. This study involving human participants was reviewed and approved by the Institutional Review Board of Southern Medical University (2022/47).

#### 2.1.2 Data analytic approach

We used R (4.4.1) for computing descriptive statistics, correlation analyses, and used Mplus Version 8.7 to carry out path analyses using the maximum likelihood (ML) method. In the model, positive metacognitive beliefs, attentional focusing and attentional shifting were entered as predictors, depressive symptoms as the outcome variable, and rumination as mediator (controlling for age and sex). We calculated the effects of positive metacognitive beliefs and attentional control (focusing, shifting) on rumination (*a* path) and depression (*c* path), effect of rumination on depression (*b* path) and indirect effects of positive metacognitive beliefs and attentional control through rumination (*ab* path).

### 2.2 Results

Descriptive statistics, reliabilities, and zero-order correlation coefficients for all the study variables are shown in **Table 1**. We found positive and significant correlations in the expected direction between all the variables of interest, indicating that individuals with impaired attentional control and higher levels of positive metacognitive beliefs about rumination showed higher levels of rumination, depressive symptoms. Attentional control and positive metacognitive beliefs were also statistically significantly correlated.

**Table 1:** Means, standard deviations, and correlations among variables of interest in cross-sectional study (N = 329).

Variables	1	2	3	4	5
1.focusing					
2.shifting	0.56***				
3.beliefs	-0.23***	-0.15*			
4.rumination	-0.58***	-0.45***	0.46***		
5.depression	-0.32***	-0.18**	0.05	0.41***	
M	21.38	20.18	23.78	47.71	12.49
SD	3.83	3.87	5.62	13.30	6.26

*p < 0.05; **p < 0.01; ***p < 0.001.

**Figure 2:**
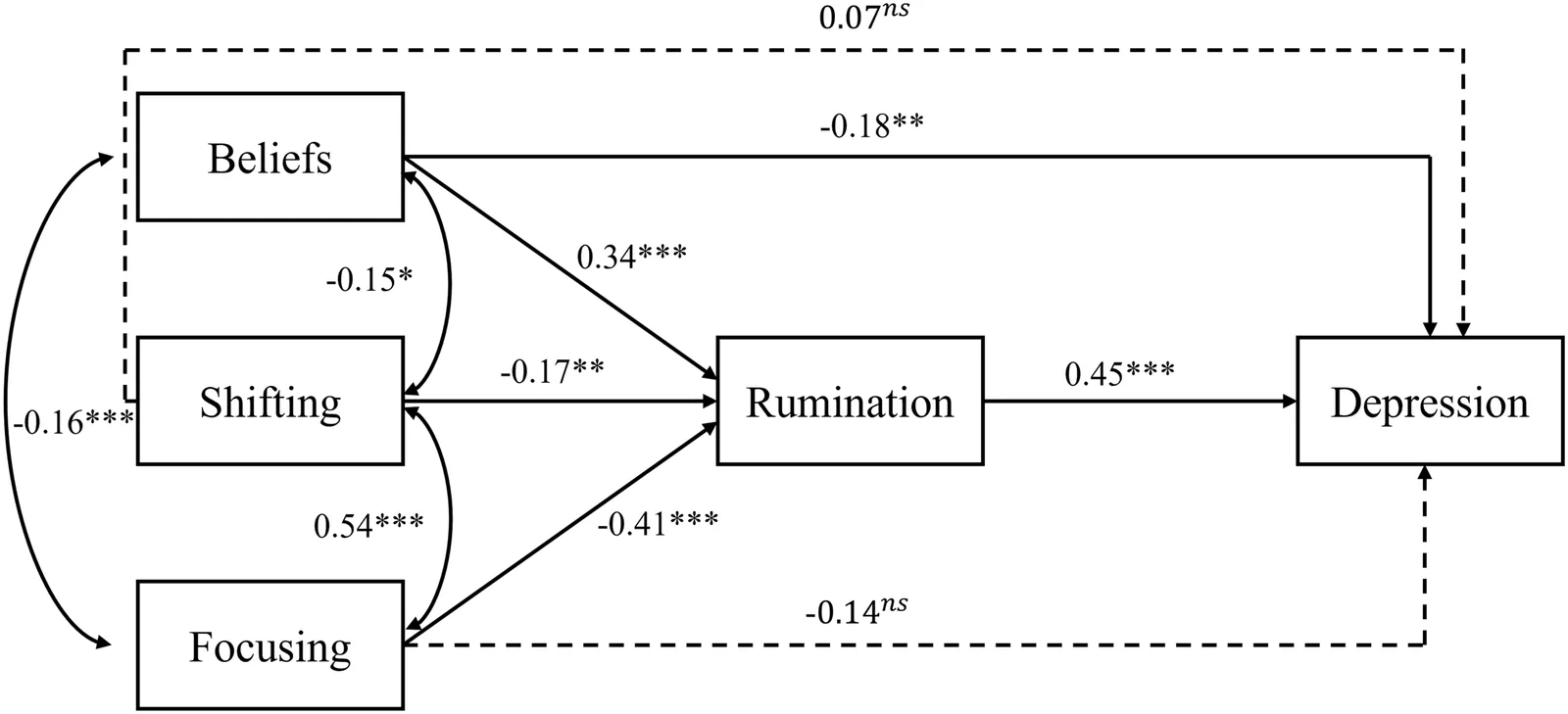
Path analysis testing rumination as a mediator among attentional control, positive metacognitive beliefs, and depression. Dashed paths represent non-significant associations. Standardized beta coefficients are shown. *p < 0.05; **p < 0.01; ***p < 0.001.

We tested the proposed mediation model, which showed the following fit indices, χ^2^ = 3.98, df = 4, *p* = 0.41; RMSEA = 0.00, 90% CI [0.00-0.08]; CFI = 1.00; SRMR = 0.03. Globally, these indices indicate a good fit to the data. As presented in **Figure 2**, attentional focusing and attentional shifting were both significantly and negatively related to rumination, while positive metacognitive beliefs was significantly and positively correlated with rumination. We also found that rumination had a significant direct effect on depression symptomatology.

For attentional focusing, the total effect was -0.53 (95% CI [-0.76 to -0.28]), the direct effect was not significant (*c*′ = -0.22, 95% CI [-0.47 to 0.02]), and the indirect effect through rumination was significant (*ab* = -0.30, 95% CI [-0.44 to -0.20]). For attentional shifting, the total effect was -0.29 (95% CI [-0.49 to -0.10]), the direct effect was not significant (*c*′ = 0.11, 95% CI [-0.08 to 0.33]), and the indirect effect through rumination was significant (*ab* = -0.40, 95% CI [-0.57 to -0.26]). For positive metacognitive beliefs, the total effect was 0.06 (95% CI [-0.08 to 0.19]), the direct effect was significant (*c*′ = -0.20, 95% CI [-0.32 to -0.06]), and the indirect effect through rumination was significant (*ab* = 0.26, 95% CI [0.18–0.35]).

### 2.3 Summary

The present cross-sectional study provided empirical support for the model in which attentional focusing, attentional shifting, and positive metacognitive beliefs contribute to depressive symptoms via rumination, corroborating previous research (24). The significant negative associations with rumination suggested that individuals with a greater self-reported capacity to maintain focus and flexibly shift attention are better equipped to disengage from ruminative thinking. This result was consistent with previous research (23, 36). Conversely, positive metacognitive beliefs were identified as a significant vulnerability factor, primarily influencing depressive symptomatology through the amplification of ruminative processes (28). This pattern highlighted the paradoxical role of metacognitive appraisal in depression—where individuals’ conviction in the functional utility of rumination (e.g., as a problem-solving strategy) inadvertently reinforces maladaptive cognitive patterns, thereby potentially sustaining self-perpetuating cycles of negative affect (26).

## 3 Study 2

### 3.1 Method

#### 3.1.1 Participants and measures

To investigate whether these dual-pathway mechanisms operate dynamically within individuals, 144 participants were recruited by posting psychological experiment notices at Southern Medical University. Firstly, we gathered the participants for a baseline measurement, which was consistent with the assessments completed by participants in Study 1. Subsequently, we carried out a 14-day EMA assessment with participants, during which they completed evaluations on attention control, rumination, depressed mood, and positive metacognitive beliefs. Each participant was assessed five times a day, starting from 10:00 AM to 10:00 PM (10:00; 13:00; 16:00; 19:00; 22:00), with assessments occurring every three hours. We excluded 9 individuals’ data using a conservative measure of noncompliance on the EMA assessment. This resulted in a final sample of 135 participants (101 female; mean age = 21.64, SD = 2.00). This study involving human participants was reviewed and approved by the Institutional Review Board of Southern Medical University (2022/47). All participant data were anonymized and financial compensation awarded to all participants upon completion of the study protocol.

The EMA items assessed depressed mood, positive metacognitive beliefs, attentional control (focusing and shifting), and rumination. Each item was rated on a visual analog scale ranging from 0 (“not at all”) to 100 (“extremely”). The exact wordings of these adapted items, their theoretical sources, and extensive empirical evidence supporting their state level reliability and validity are detailed comprehensively in the **Supplementary Material** (see Measurement scales).

#### 3.1.2 Data analytic approach using DSEM

This study employed DSEM (37) in Mplus Version 8.7 to analyze intensive longitudinal data collected over 14 days, with 5 daily time points (70 observations per subject), nested within individuals (Level 2). Two Markov chains of 20,000 iterations each were executed, with the first 50% discarded as burn-in to ensure stationarity. Convergence was rigorously monitored using the posterior scale reduction (PSR) statistic, requiring PSR < 1.1 for all parameters. Diffuse priors were specified per Mplus defaults. Parameter estimates derived from posterior medians were evaluated using 95% highest posterior density (HPD) intervals, with effects deemed significant when intervals excluded zero. To fully disaggregate the between-person and within-person variances, the DSEM framework was applied to specify a multilevel dynamic mediation model. Specifically, at the within-person level, the mediating role of rumination between attentional control (focusing and shifting), positive metacognitive beliefs and depressed mood were tested (38).

We first examined contemporaneous (*t*) associations among attentional shifting, attentional focusing, positive metacognitive beliefs, rumination, and depressed mood within the DSEM framework. For the mediation, the effect of attentional focusing on same timepoint rumination represents *a*-path, and the effect of rumination on same timepoint depressed mood represents *b*-path. The conditional direct effect of attentional focusing on same timepoint depressed mood represents *c*′-path. Concurrently, we estimated two additional models within the DSEM framework, only replacing attentional focusing with attentional shifting and positive metacognitive beliefs, while everything else remained identical.

Next, we estimated lagged (*t*-1) effects to capture dynamic, time-varying processes. For the mediation, the effect of attentional focusing on same timepoint rumination represents *a*-path, and the effect of rumination on next timepoint depressed mood represents *b*-path. The conditional direct effect of attentional focusing on next timepoint depressed mood represents *c*′-path. Likewise, two additional lagged models were estimated by replacing attentional focusing with attentional shifting and positive metacognitive beliefs, respectively, while keeping all other paths identical.

In addition, for theoretical completeness, we explored higher-order lagged effects (e.g., *t*-2→*t*-1→*t*). Except for the model specifying positive metacognitive beliefs as the predictor, which demonstrated sustained temporal paths, these higher-order models generally did not yield statistically significant results. These exploratory analyses are reported in the **Supplementary Materials** to comprehensively delineate the duration of the lingering effects over time and to guide future research employing denser sampling schedules (see supplementary analysis of higher-order lagged effects model).

For all the models specified above, all variables were latent person-mean centered at the within-person level to isolate within-person variances. Furthermore, all path coefficients were estimated while fully controlling for the first-order autoregressive effects of each respective variable (see **Supplementary Materials** for additional model details).

### 3.2 Results

#### 3.2.1 Descriptive statistics and correlations

Descriptive statistics and bivariate correlations for all study variables are presented in **Table 2**. Correlational analyses revealed significant relationships consistent with the disengagement impairment hypothesis. Both attention control components showed strong negative associations with rumination and depression symptoms. Positive metacognitive beliefs about rumination were significantly positively correlated with rumination. Critically, rumination demonstrated a strong positive association with depression symptoms, consistent with its hypothesized mediating role. The attention control variables were moderately intercorrelated, while positive metacognitive beliefs showed a small negative association with focusing but not with shifting. Notably, this correlational pattern was largely consistent with the results observed in Study 1, reinforcing the robustness of these associations across independent cohorts.

**Table 2:** Means, standard deviations, and correlations among variables of interest.

Variables	1	2	3	4	5
1.focusing					
2.shifting	0.49***				
3.beliefs	-0.21*	-0.11			
4.rumination	-0.49***	-0.45***	0.44***		
5.depression	-0.43***	-0.28***	0.13	0.69***	
M	20.90	19.70	24.44	50.30	8.13
SD	3.65	3.64	5.60	13.30	5.23

*p < 0.05; ***p < 0.001.

#### 3.2.2 Contemporaneous mediation by rumination between attentional focusing and depressed mood

**Figure 3:**
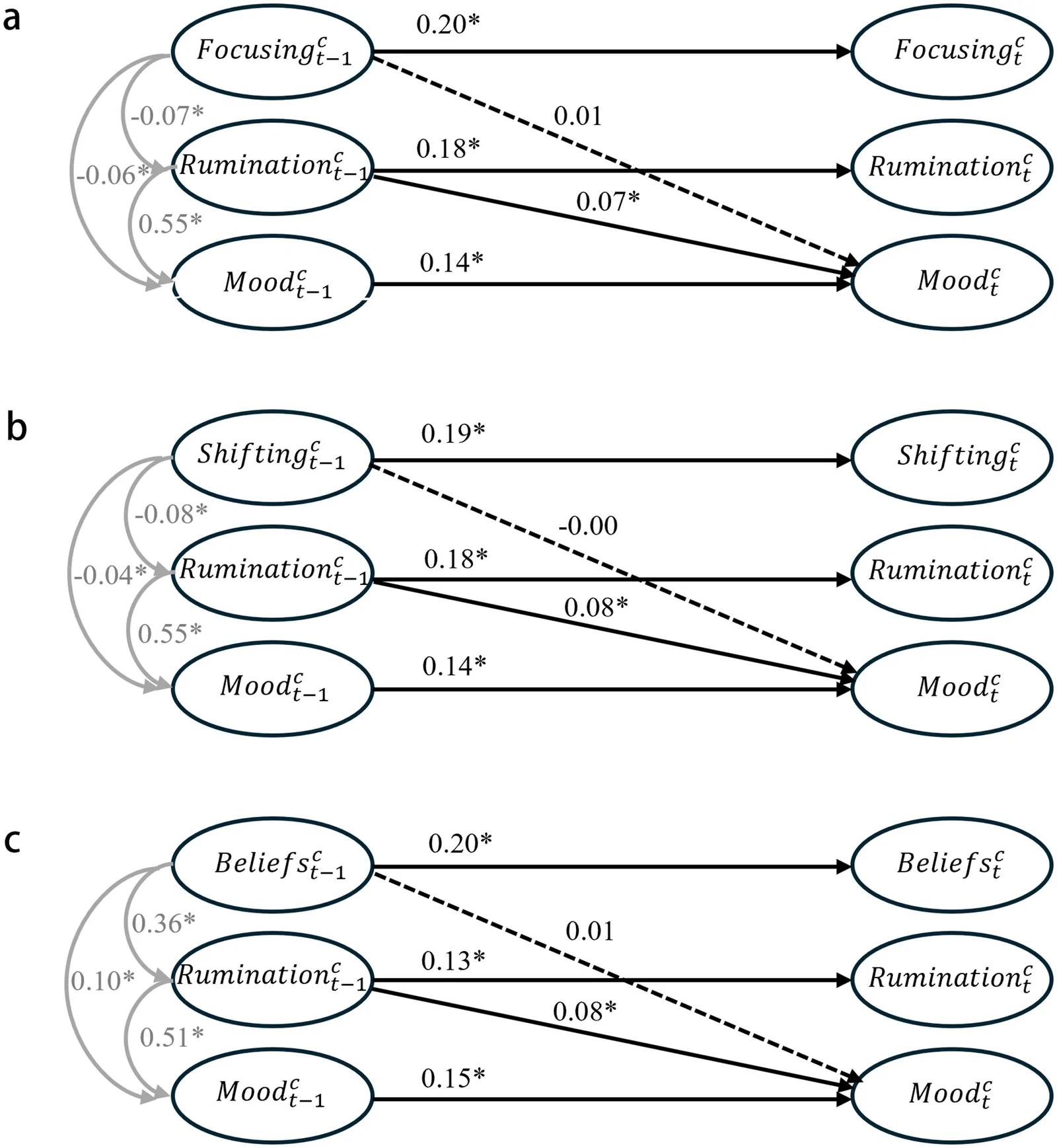
Both contemporaneous and lagged within-person mediation model. Solid gray pathways represent results from the contemporaneous DSEM. Solid black pathways represent results from the first-order lagged DSEM. **(a)** Within-person mediation model for attentional focusing, rumination, and depressed mood. **(b)** Within-person mediation model for attentional shifting, rumination, and depressed mood. **(c)** Within-person mediation model for positive metacognitive beliefs, rumination, and depressed mood.

In the contemporaneous model with attentional focusing as the predictor, attentional focusing significantly reduced rumination (*a* = -0.07, 95% HPD CI [-0.12, -0.02]) and directly lowered depressed mood (*c*′= -0.06, 95% HPD CI [-0.08, -0.03]), whereas rumination robustly exacerbated depressed mood (*b* = 0.55, 95% HPD CI [0.51, 0.58]), with 95% highest posterior density (HPD) credible intervals excluding zero (see **Figure 3a**). The average mediation effect was significant (non-zero; E (*a_j_b_j_*) = -0.04, 95% HPD CI [-0.07, -0.01]). Comprehensive details for all parameter estimates are provided in the **Supplementary Materials** (**Supplementary Table 2**).

#### 3.2.3 Contemporaneous mediation by rumination between attentional shifting and depressed mood

Significant results were revealed in the model where attentional shifting served as the predictor. (*a* = -0.08, 95% HPD CI [-0.13, -0.03]; *b* = 0.55, 95% HPD CI [0.52, 0.59]; *c*′ = -0.04, 95% HPD CI [-0.07, -0.02]; **Figure 3b**). The average mediation effect was significant (non-zero; E (*a_j_b_j_*) = -0.04, 95% HPD CI [-0.07, -0.02]). Comprehensive details for all parameter estimates are provided in **Supplementary Table 3**.

#### 3.2.4 Contemporaneous mediation by rumination between positive metacognitive beliefs and depressed mood

The model specifying positive metacognitive beliefs as the predictor revealed that it significantly and positively predicted both rumination (a = 0.36, 95% HPD CI [0.32, 0.41]) and depressed mood (*c*′ = 0.10, 95% HPD CI [0.07, 0.13]), whereas rumination robustly exacerbated depressed mood (b = 0.51, 95% HPD CI [0.48, 0.55]) (see **Figure 3c**). The average mediation effect was significant (non-zero; E (*a_j_b_j_*) = 0.19, 95% HPD CI [0.16, 0.21]). Comprehensive details for all parameter estimates are provided in **Supplementary Table 4**.

#### 3.2.5 Lagged mediation by rumination between attentional focusing and depressed mood

We fitted this model with the Bayesian Markov chain Monte Carlo estimation. All parameters except for the direct effect *c*′ and the latent means (*l_X_*, *l_M_*, *l_Y_*) demonstrated statistical significance, with 95% highest posterior density (HPD) credible intervals excluding zero (see **Supplementary Table 5**). Significant results were revealed in the model where attentional focusing served as the predictor. (*a* = -0.07, 95% HPD CI [-0.12, -0.02]; *b* = 0.07, 95% HPD CI [0.03, 0.11]; **Figure 3a**). The average mediation effect was significant (non-zero; E (*a_j_b_j_*) = -0.005, 95% HPD CI [-0.010, -0.001]).

#### 3.2.6 Lagged mediation by rumination between attentional shifting and depressed mood

All parameters except for the direct effect *c*′ and the latent means (*l_X_*, *l_M_*, *l_Y_*) demonstrated statistical significance (see **Supplementary Table 6**). Significant results were revealed in the model where attentional shifting served as the predictor. (*a* = -0.08, 95% HPD CI [-0.13, -0.03]; *b* = 0.08, 95% HPD CI [0.04, 0.12]; **Figure 3b**). The average mediation effect was significant (non-zero; E (*a_j_b_j_*) = -0.006, 95% HPD CI [-0.011, -0.002]).

#### 3.2.7 Lagged mediation by rumination between positive metacognitive beliefs and depressed mood

All parameters except for the direct effect *c*′ and the latent means (*l_X_*, *l_M_*, *l_Y_*) demonstrated statistical significance (see **Supplementary Table 7**). Significant results were revealed in the model where positive metacognitive beliefs served as the predictor. (*a* = 0.36, 95% HPD CI [0.32, 0.40]; *b* = 0.08, 95% HPD CI [0.04, 0.12]; **Figure 3c**). The average mediation effect was significant (non-zero; E (*a_j_b_j_*) = 0.029, 95% HPD CI [0.014, 0.044]).

### 3.3 Summary

The results in study 2 confirmed the existence of three distinct dynamic pathways through which attentional focusing, attentional shifting, and positive metacognitive beliefs influence depressed mood via rumination within both contemporaneous (*t*) and first-order lagged (*t*-1) models. Specifically, after controlling for the autoregressive effect of rumination, both attentional focusing and attentional shifting significantly and negatively predicted rumination at the same time point, while positive metacognitive beliefs significantly and positively predicted rumination. Furthermore, rumination significantly predicted the increases in depressed mood at both the current and subsequent time points.

## 4 General discussion

This study integrated cross-sectional and intensive longitudinal designs to investigate the mechanisms through which rumination contributes to the maintenance of depression. In Study 1, the mediating role of rumination between attentional control, positive metacognitive beliefs, and depression was examined. The results indicated that both attentional shifting and focusing contributed to depressive symptomatology through the mediational role of rumination. Aligning with the findings of DeJong et al. (19), which demonstrated that rumination mediated the associations of both self-reported attentional focusing and shifting with depressive symptoms. Besides, rumination mediated the relations between positive metacognitive beliefs and depression. The current findings support recently proposed models of rumination (15, 24, 39).

In Study 2, EMA and DSEM were utilized to examine the within-person dynamics among these cognitive variables. By modeling the three pathways separately, this study aimed to examine the independent dynamic pathways through which each cognitive component influences mood via rumination. Specifically, the study investigated both contemporaneous and lagged associations between the variables of interest to identify their dynamic pathways. In the models (attentional control as predictor), results showed that both attentional shifting and focusing predicted rumination at the same time-point. The contemporaneous predictive effect of attentional control on rumination has been documented in previous research (31), which is also consistent with the self-regulatory theories of rumination that have assigned a unique role to attentional control deficits in the initiation and maintenance of ruminative states (3, 40). This finding further extends the relationship between attentional control and repetitive negative thinking to the state level, whereas previous research has predominantly focused on its trait-level manifestations (23). Alternatively, after controlling for depressed mood at the previous time-point, rumination predicted depressed mood at both the concurrent and subsequent time-points, which aligns with theories conceptualizing rumination as a cognitive load that depletes emotional resources and prolongs negative affect beyond the initial triggering context (41, 42). These findings support the role of rumination in the emergence and maintenance of depressed mood, consistent with prior research that characterizes rumination as a maladaptive emotion regulation strategy which contributes to the increase of negative affect (43). Furthermore, a recent study found that rumination is associated with prolonged impairment of mood (44).

Both attentional shifting and focusing significantly predicted depressed mood at the concurrent time point, yet neither predicted depressed mood at the subsequent time point. These findings indicate that the buffering effect of state attentional control against depressed mood is subject to temporal constraints, potentially implying that future clinical applications might need to consider specific timing windows when integrating cognitive training into daily life. In both contemporaneous and first-order lagged models, rumination significantly mediated the relationship between attentional control (both attentional shifting and focusing) and depressed mood. The mediating role of rumination between attentional control and depressed mood offers preliminary evidence that may have implications for transdiagnostic processes, although this possibility requires robust validation in clinical and clinically heterogeneous samples (45). These findings demonstrate that both attentional focusing and shifting were associated with lower depressed mood by being linked to reduced concurrent rumination, and furthermore extended their buffering implication against depressed mood at the subsequent assessment through this same pathway of rumination reduction. Alternatively, These findings are consistent with therapeutic approaches that target attentional control to reduce rumination and subsequently improve mood, such as mindfulness-based cognitive therapy or alternative physical interventions (46–48).

In the models (positive metacognitive beliefs as predictor), results demonstrated that positive metacognitive beliefs significantly predicted rumination at both the concurrent and subsequent time points after controlling the rumination at previous time-point. This indicates that positive metacognitive beliefs activate and maintain rumination, aligning with the proposition of metacognitive model that positive metacognitive beliefs activate the use of metacognitive strategies such as rumination (25, 26, 49). Furthermore, the study also found that the mediating role of rumination between positive metacognitive beliefs and depressed mood was significant not only in contemporaneous and first-order lagged models, but also in higher-order lagged models (see supplementary analysis of higher-order lagged effects model). Notably, the results from the higher-order lagged models indicate that positive metacognitive beliefs still exerted an influence on depressed mood at the second subsequent assessment by sustaining rumination. These findings align with the results of Cano-López et al. (32), which showed that metacognitive strategies mediated the relations between metacognitive beliefs and negative affect within days. Our results provide temporally ordered, within-person evidence for this relationship. Indeed, this study not only provide support for metacognitive therapy (50, 51), but also offer richer empirical and theoretical foundations for the role of positive metacognitive beliefs in improving rumination and depression. For example, the efficacy of negative metacognitive beliefs has confirmed in metacognitive therapy, whereas the role of positive metacognitive beliefs remains unclear (52).

The current study investigates the within-person dynamics of attentional control, positive metacognitive beliefs, rumination, and depressed mood, providing evidence consistent with temporally ordered processes among these variables. The within-person lagged effects observed here offer a more granular view than between-person designs alone, though it should be acknowledged that the three-hour assessment interval may not fully capture more rapid dynamics such as momentary attentional disengagement or quick affective shifts; the attenuation of certain lagged effects may partly reflect the spacing of assessments. With denser sampling schedules, future studies could more precisely delineate the timescale of these cognitive processes. Moreover, the temporally ordered pathways identified here could be examined under rigorous experimental designs to formally evaluate causality. Beyond these methodological considerations, our research provides proximal dynamic evidence regarding potential transdiagnostic processes related to depressed mood in a non-clinical sample, which may offer useful insights for both theoretical models and the future development of temporally targeted intervention frameworks.

## 5 Limitations

Several limitations warrant consideration. First, both studies are observational. Although EMA-based DSEM provides temporally ordered within-person evidence, unmeasured time-varying confounders (e.g., daily stressors) may still influence the estimated pathways. Second, Study 2 examined the three cognitive pathways in separate rather than simultaneous models, precluding assessment of their interactive effects within an integrated framework. Third, the five-assessment-per-day schedule, while feasible, may be too sparse to fully capture rapid cognitive processes such as attentional disengagement and affective shifts. Future studies with denser sampling (e.g., event-contingent or sub-hourly designs) could provide finer temporal resolution, which would help determine whether the attenuation of lagged effects observed here reflects genuine temporal boundaries of cognitive processes or an artifact of assessment spacing, and more precisely delineate the timescale over which attentional control and metacognitive beliefs influence rumination and mood in daily life. Fourth, the EMA measures relied on self-report items adapted for state-level assessment rather than validated multi-item scales or objective behavioral tasks. Using concise items to capture complex cognitive constructs such as attentional control and metacognition in daily life is inherently challenging, and formal psychometric validation of these state-level single items was not conducted. This may introduce both common-method variance and construct-validity concerns. Future studies could complement self-report EMA items with cognitive experimental tasks to validate and strengthen the robustness of the observed dynamic associations. Fifth, generalizability is constrained by the predominantly student sample, the absence of clinical diagnostic assessment, and the smartphone-based EMA protocol. The findings in this non-clinical sample should not be extrapolated to populations diagnosed with major depressive disorder without further research. Sixth, no formal priori power analysis was conducted for the intensive longitudinal design prior to data collection. Although our intensive sampling scale (N = 135 with 70 observations per participant) satisfies contemporary precision and parameter convergence recommendations for stable DSEM estimation based on prior simulation guidelines (53), this methodological constraint should be considered when interpreting the statistical boundaries of our findings.

## 6 Conclusions

This study conducted a multi-method investigation into the within-person, time-varying cognitive mechanisms associated with depressed mood through rumination. Specifically, we demonstrated that both attentional control deficits and positive metacognitive beliefs predict increased rumination, which in turn is linked to the exacerbation of depressive symptoms. We further confirmed the existence of three dynamic pathways in which attentional focusing, attentional shifting, and positive metacognitive beliefs influence depressed mood via rumination. These findings may provide a useful framework to inform future studies testing precisely timed or just-in-time adaptive interventions that target cognitive vulnerabilities in daily life.

## Data Availability

The raw data supporting the conclusions of this article will be made available by the authors, without undue reservation.
