# Supplementary material for: Temporal dynamics of rumination in daily life: a multi-method investigation of attentional control and metacognitive pathways toward depression
Source: Front Psychiatry. 2026 Aug 17;17:1900459. doi: 10.3389/fpsyt.2026.1900459 (PMC13528211; doi:10.3389/fpsyt.2026.1900459)
Supplement: Supplementary file 1 [file Table1.docx]

Supplementary Material

**Temporal Dynamics of Rumination in Daily Life: A Multi-Method Investigation of Attentional Control and Metacognitive Pathways toward Depression**

**Contents**

[Measurement scales	1](#_Toc234590702)

[A presentation of all the DSEM details	3](#_Toc234590703)

[Supplementary tables with parameter estimates	6](#_Toc234590704)

[Supplementary analysis of higher-order lagged effects model (from *t-2* to *t-1* to *t*)	11](#_Toc234590705)

[Supplementary references	16](#_Toc234590706)

**Measurement scales**

**Study 1**

**Attentional Control.** The Attentional Control Scale (Derryberry & Reed, 2002), is a self-report scale aiming to assess executive control over attention. We used the Chinese version of the ACS (He, 2021), revised from the original scale developed by Derryberry, consists of 16 items across two factors. The Attention Focusing subscale (items 1-8) assesses the inhibitory function of cognitive control, while the Attention Shifting subscale (items 9-16) evaluates the shifting function of cognitive control. All items use a 4-point Likert scale, with responses ranging from 1 (“almost never”) to 4 (“always”). Total scores range from 16 to 64, with higher scores indicating stronger attentional control abilities.

**Positive Beliefs about Rumination Scale**. PBRS (Papageorgiou & Wells, 2001) is a 9-item self-report scale that assesses positive metacognitive beliefs about rumination. Respondents are asked to indicate the extent to which they agree with each item on a 4-Likert scale ranging between 1 = “do not agree” and 4 = “agree very much”. The total score ranges from 9 to 36, with higher scores indicating greater conviction in positive metacognitive beliefs. The Chinese version we used have shown good reliability and validity (Zhou et al., 2023).

**Ruminative Responses Scale (RRS)**. The RRS (Nolen-Hoeksema & Morrow, 1991) is a 22-item self-report scale that assesses individuals’ tendencies to ruminate in response to sadness or depressed mood. It is composed of three factors: symptom rumination, brooding, and reflective pondering. Participants rate each item on a four - point Likert scale, with 1 indicating “almost never” and 4 representing “almost always”. Higher total scores on the RRS suggest more severe depressive rumination. Both the original version and the Chinese version we used have shown good reliability and validity (Han & Yang, 2009; Nolen-Hoeksema & Morrow, 1991).

**Patient Health Questionnaire-9 (PHQ-9)**. The PHQ-9 is a self-report tool developed to measure the severity of depressive symptoms (Kroenke et al., 2001). It is based on the nine diagnostic criteria for major depressive disorders in the Diagnostic and Statistical Manual of Mental Disorders (DSM). Respondents rate how often they experience symptoms such as “little interest or pleasure in doing things” over the past two weeks on a 4-point scale, from 0 (“not at all”) to 3 (“nearly every day”). The total score, ranging from 0 to 27, is calculated by summing the item responses, with higher scores indicating more severe depressive symptoms. Both the original version and the Chinese version we used have shown good reliability and validity (Kroenke et al., 2001; Wang et al., 2014).

**Study 2**

**Depressed mood.** The depressed mood assessment includes one item: “At this moment, I feel down, depressed, or hopeless.” This item is derived directly from the second symptom of the PHQ-9, in which it serves as the core indicator for assessing depressed mood (Kroenke et al., 2001).

**Positive metacognitive beliefs.** The assessment includes one item: “In the past three hours, ruminating about the past and my problems help me to find answers for my current depressed mood.” This item was adapted from the validated Positive Beliefs About Rumination Scale (Papageorgiou & Wells, 2001). Our adaptation retains the core construct while framing it as a state-like assessment suitable for EMA.

**Attentional control.** The attentional state assessment includes two items adapted from the Chinese version of the Attentional Control Scale (ACS-C). The shifting item (“In the past three hours, when distracting thoughts arose, I was able to easily bring my attention back.”) was adapted from Item 15 of the ACS-C, which belongs to the attentional shifting subscale. The focusing item (“In the past three hours, when focusing on a task, I was able to concentrate and not be distracted by unrelated thoughts.”) was adapted from Item 7 of the ACS-C (originally a reverse-scored item) and reframed in positive wording to directly assess the ability to maintain attentional focus.

**Rumination.** The rumination assessment includes one item: “In the past three hours, the same negative thoughts keep going through my mind again and again.” This item used to assess rumination was based on the validated process-oriented paradigm developed by Müller et al. (2025). Their research demonstrated that EMA-based assessment of repetitive negative thinking process characteristics (e.g., repetitiveness, intrusiveness) showed high reliability and predictive validity for future psychopathology. Our single-item measure focused specifically on the dimension of repetitiveness, which is central to the component of rumination and was a key component of their validated model.

**A presentation of all the DSEM details**

We implemented Bayesian Markov Chain Monte Carlo estimation in Mplus 8.7 to analyze dynamic structural equation models, adhering to methodological standards for intensive longitudinal data (McNeish & MacKinnon, 2022). Two Markov chains of 20,000 iterations each were executed, with the first 50% discarded as burn-in to ensure stationarity. Convergence was rigorously monitored using the posterior scale reduction (PSR) statistic, requiring PSR < 1.1 for all parameters. Diffuse priors were specified per Mplus defaults. Parameter estimates derived from posterior medians were evaluated using 95% highest posterior density (HPD) intervals, with effects deemed significant when intervals excluded zero.

***Contemporaneous DSEM (***$\boldsymbol{X}_{\boldsymbol{tj}}$***→***$\boldsymbol{M}_{\boldsymbol{tj}}$***→***$\boldsymbol{Y}_{\boldsymbol{tj}}$***)***

For a three-variable mediation model with an independent variable $X_{tj}$ (i.e., *X* at Time *t* for Individual *j*), a mediator $M_{tj}$, and a dependent variable $Y_{tj}$, the 1-1-1 intensive longitudinal mediation (ILM) model can be specified as

Level 1:

$$\begin{aligned} X_{tj}^{c}=\beta_{Xj}X_{\left( t-1 \right)j}^{c}+\varepsilon_{Xjt},\#\left( 1 \right) \end{aligned}$$

$$\begin{aligned} M_{tj}^{c}=\beta_{Mj}M_{\left( t-1 \right)j}^{c}+a_{j}X_{tj}^{c}+\varepsilon_{Mjt},\#\left( 2 \right) \end{aligned}$$

$$\begin{aligned} Y_{tj}^{c}=\beta_{Yj}Y_{\left( t-1 \right)j}^{c}+b_{j}M_{tj}^{c}+c_{j}^{'}X_{tj}^{c}+\varepsilon_{Yjt},\#\left( 3 \right) \end{aligned}$$

Level 2:

$$\begin{aligned} l_{Xj}=l_{X}+\mu_{1j}, l_{Mj}=l_{M}+\mu_{2j}, l_{Yj}=l_{Y}+\mu_{3j},\#\left( 4 \right) \end{aligned}$$

$$\begin{aligned} \beta_{Xj}=\beta_{X}+\mu_{4j}, \beta_{Mj}=\beta_{M}+\mu_{5j}, \beta_{Yj}=\beta_{Y}+\mu_{6j},\#\left( 5 \right) \end{aligned}$$

$$\begin{aligned} a_{j}=a+\mu_{7j}, b_{j}=b+\mu_{8j}, c_{j}^{'}=c_{j}+\mu_{9j},\#\left( 6 \right) \end{aligned}$$

$$\begin{aligned} \log\left( \sigma_{Xj}^{2} \right)=\omega_{X}+\mu_{10j}, \log\left( \sigma_{Mj}^{2} \right)=\omega_{M}+\mu_{11j}, \log\left( \sigma_{Yj}^{2} \right)=\omega_{Y}+\mu_{12j},\#\left( 7 \right) \end{aligned}$$

where $X_{tj}^{c}$, $M_{tj}^{c}$, and $Y_{tj}^{c}$ with a superscript “*c*” denoting these variables are centered at latent individual-mean $l_{Xj}$, $l_{Mj}$, and $l_{Yj}$. That is:

$$\begin{aligned} X_{tj}^{c}=X_{tj}-l_{Xj}, M_{tj}^{c}=M_{tj}-l_{Mj}, Y_{tj}^{c}=Y_{tj}-l_{Yj},\#\left( 8 \right) \end{aligned}$$

The $\beta_{Xj}$, $\beta_{Mj}$, and $\beta_{Yj}$ coefficients represent first-order (i.e., lagged-1) autoregressive effects because the variable at occasion (time) *t* is predicted by its prior occasion *t*-1 value. The $a_{j}$, $b_{j}$, and $c_{j}^{'}$ coefficients represent the immediate effects. It's worth mentioning that $\beta_{Xj}$, $\beta_{Mj}$, $\beta_{Yj}$, $a_{j}$, $b_{j}$, and $c_{j}^{'}$ do not have the subscript *t*, meaning that we assume the autoregressive and immediate effects do not vary over time in DSEM. For Level 2, the $l_{Xj}$, $l_{Mj}$, $l_{Yj}$, $\beta_{Xj}$, $\beta_{Mj}$, $\beta_{Yj}$, $a_{j}$, $b_{j}$, and $c_{j}^{'}$ coefficients can be specified as a random effect. The total average mediation effect was defined as *ab*, excluding the $\sigma_{a_{j}b_{j}}$(see Fig.S1) (McNeish & MacKinnon, 2022).

$$\begin{aligned} E\left( a_{j}b_{j} \right)=ab,\#\left( 9 \right) \end{aligned}$$

***First-order lagged DSEM (***$\boldsymbol{X}_{\left( \boldsymbol{t-1} \right)\boldsymbol{j}}$***→***$\boldsymbol{M}_{\left( \boldsymbol{t-1} \right)\boldsymbol{j}}$***→***$\boldsymbol{Y}_{\boldsymbol{tj}}$***)***

Consistent with the contemporaneous ($X_{tj}$→$M_{tj}$→$Y_{tj}$) DSEM, these variables are centered at latent individual-mean $l_{Xj}$, $l_{Mj}$, and $l_{Yj}$. Similarly, at Level 2, adjustments are made to the temporal order of the relationships among Level 1 variables, in line with the contemporaneous DSEM. In this case, $a_{j}$ represents the immediate effect of *X* on *M*, $b_{j}$ represents the lagged-1 effect of *M* on *Y* and $c_{j}^{'}$ represent lagged-1 effect of *X* on *M* (see Fig.S1). The equations of Level 1 can be specified as

Level 1:

$$\begin{aligned} X_{tj}^{c}=\beta_{Xj}X_{\left( t-1 \right)j}^{c}+\varepsilon_{Xjt},\#\left( 10 \right) \end{aligned}$$

$$\begin{aligned} M_{tj}^{c}=\beta_{Mj}M_{\left( t-1 \right)j}^{c}+a_{j}X_{tj}^{c}+\varepsilon_{Mjt},\#\left( 11 \right) \end{aligned}$$

$$\begin{aligned} Y_{tj}^{c}=\beta_{Yj}Y_{\left( t-1 \right)j}^{c}+b_{j}M_{\left( t-1 \right)j}^{c}+c_{j}^{'}X_{\left( t-1 \right)j}^{c}+\varepsilon_{Yjt}.\#\left( 12 \right) \end{aligned}$$

*Insert Figure S1 here*

**Figure S1.** Schematic representation of the 1-1-1 intensive longitudinal mediation (ILM) model based on DSEM. The paths illustrate the dynamic relationships for two distinct DSEM models fitted to the same data. Solid grey pathways represent results from the contemporaneous DSEM ($X_{tj}$→$M_{tj}$→$Y_{tj}$), where all variables are assessed at the same point t. Solid black pathways represent results from the first-order lagged DSEM ($X_{\left( t-1 \right)j}$→$M_{\left( t-1 \right)j}$→$Y_{tj}$).

Table S1. The DIC and final PSR after 20000 iterations for DSEM.

| Model | PSR | DIC |
| --- | --- | --- |
| ${Focusing}_{tj}$*→*${Rumination}_{tj}$*→*${Depression}_{tj}$ | 1.018 | 72918 |
| ${Shifting}_{tj}$*→*${Rumination}_{tj}$*→*${Depression}_{tj}$ | 1.026 | 73111 |
| ${Beliefs}_{tj}$*→*${Rumination}_{tj}$*→*${Depression}_{tj}$ | 1.034 | 71463 |
| ${Focusing}_{\left( t-1 \right)j}$*→*${Rumination}_{\left( t-1 \right)j}$*→*${Depression}_{tj}$ | 1.015 | 77110 |
| ${Shifting}_{\left( t-1 \right)j}$*→*${Rumination}_{\left( t-1 \right)j}$*→*${Depression}_{tj}$ | 1.034 | 77270 |
| ${Beliefs}_{\left( t-1 \right)j}$*→*${Rumination}_{\left( t-1 \right)j}$*→*${Depression}_{tj}$ | 1.016 | 75750 |
| ${Focusing}_{\left( t-2 \right)j}$*→*${Rumination}_{\left( t-1 \right)j}$*→*${Depression}_{tj}$ | 1.035 | 77491 |
| ${Shifting}_{\left( t-2 \right)j}$*→*${Rumination}_{\left( t-1 \right)j}$*→*${Depression}_{tj}$ | 1.041 | 77726 |
| ${Beliefs}_{\left( t-2 \right)j}$*→*${Rumination}_{\left( t-1 \right)j}$*→*${Depression}_{tj}$ | 1.042 | 77538 |

**Supplementary tables with parameter estimates**

Table S2. Parameter estimates and HPD CI for all parameters of the contemporaneous (${Focusing}_{tj}$→${Rumination}_{tj}$→${Depression}_{tj}$) Dynamic Structural Equation Model with attentional focusing as the predictor.

| Par | Est. | 95% HPD CI | PSR | Ess |
| --- | --- | --- | --- | --- |
| a | -0.072 | [-0.121, -0.021] | 1.000 | 5395 |
| b | 0.547 | [0.510, 0.584] | 0.999 | 21671 |
| $c^{'}$ | -0.055 | [-0.079, -0.031] | 1.000 | 13444 |
| $\beta_{X}$ | 0.200 | [0.154, 0.244] | 1.000 | 6658 |
| $\beta_{M}$ | 0.176 | [0.133, 0.218] | 1.000 | 8214 |
| $\beta_{Y}$ | 0.074 | [0.048, 0.099] | - | - |
| $l_{X}$ | 0.000 | [-0.027, 0.024] | 1.001 | 929 |
| $l_{M}$ | -0.003 | [-0.028, 0.025] | 1.006 | 523 |
| $l_{Y}$ | -0.001 | [-0.024, 0.025] | 1.008 | 451 |
| $\omega_{X}$ | -0.111 | [-0.155, -0.066] | 1.000 | 2454 |
| $\omega_{M}$ | -0.185 | [-0.235, -0.136] | 1.003 | 620 |
| $\omega_{Y}$ | -0.574 | [-0.658, -0.488] | 1.000 | 4507 |
| ${var(\mu}_{1j})$ | 0.002 | [0.001, 0.005] |  |  |
| ${var(\mu}_{2j})$ | 0.002 | [0.001, 0.004] |  |  |
| ${var(\mu}_{3j})$ | 0.002 | [0.001, 0.004] |  |  |
| ${var(\mu}_{4j})$ | 0.056 | [0.041,0.078] |  |  |
| ${var(\mu}_{5j})$ | 0.049 | [0.035, 0.068] |  |  |
| ${var(\mu}_{6j})$ | 0.012 | [0.007, 0.019] |  |  |
| ${var(\mu}_{7j})$ | 0.073 | [0.054, 0.100] |  |  |
| ${var(\mu}_{8j})$ | 0.036 | [0.026, 0.051] |  |  |
| ${var(\mu}_{9j})$ | 0.011 | [0.006, 0.017] |  |  |
| ${var(\mu}_{10j})$ | 0.046 | [0.028, 0.071] |  |  |
| ${var(\mu}_{11j})$ | 0.055 | [0.036, 0.028] |  |  |
| ${var(\mu}_{12j})$ | 0.219 | [0.162, 0.299] |  |  |

Note. Est. = median of the posterior, HPD CI = highest posterior density credible intervals, PSR = posterior scale reduction, Ess = effective sample sizes. “-” = fixed parameter in Mplus DSEM.

Table S3. Parameter estimates and HPD CI for all parameters of the contemporaneous (${Shifting}_{tj}$→${Rumination}_{tj}$→${Depression}_{tj}$) Dynamic Structural Equation Model with attentional shifting as the predictor.

| Par | Est. | 95% HPD CI | PSR | Ess |
| --- | --- | --- | --- | --- |
| a | -0.077 | [-0.127, -0.027] | 1.000 | 3812 |
| b | 0.552 | [0.515, 0.589] | 1.000 | 24560 |
| $c^{'}$ | -0.044 | [-0.066, -0.022] | 1.000 | 15801 |
| $\beta_{X}$ | 0.189 | [0.146, 0.231] | 1.000 | 7021 |
| $\beta_{M}$ | 0.181 | [0.138, 0.223] | 1.000 | 10782 |
| $\beta_{Y}$ | 0.075 | [0.049, 0.101] | - | - |
| $l_{X}$ | 0.000 | [-0.027, 0.025] | 1.003 | 956 |
| $l_{M}$ | 0.000 | [-0.027, 0.027] | 1.003 | 481 |
| $l_{Y}$ | -0.006 | [-0.023, 0.027] | 1.000 | 451 |
| $\omega_{X}$ | -0.095 | [-0.134, -0.056] | 1.000 | 3011 |
| $\omega_{M}$ | -0.188 | [-0.237, -0.139] | 1.001 | 354 |
| $\omega_{Y}$ | -0.568 | [-0.652, -0.482] | 1.000 | 4722 |
| ${var(\mu}_{1j})$ | 0.002 | [0.001, 0.005] |  |  |
| ${var(\mu}_{2j})$ | 0.002 | [0.001, 0.005] |  |  |
| ${var(\mu}_{3j})$ | 0.002 | [0.001, 0.004] |  |  |
| ${var(\mu}_{4j})$ | 0.049 | [0.035,0.069] |  |  |
| ${var(\mu}_{5j})$ | 0.050 | [0.036, 0.070] |  |  |
| ${var(\mu}_{6j})$ | 0.013 | [0.008, 0.020] |  |  |
| ${var(\mu}_{7j})$ | 0.073 | [0.054, 0.100] |  |  |
| ${var(\mu}_{8j})$ | 0.037 | [0.027, 0.052] |  |  |
| ${var(\mu}_{9j})$ | 0.007 | [0.004, 0.012] |  |  |
| ${var(\mu}_{10j})$ | 0.026 | [0.015, 0.041] |  |  |
| ${var(\mu}_{11j})$ | 0.053 | [0.034, 0.078] |  |  |
| ${var(\mu}_{12j})$ | 0.218 | [0.161, 0.297] |  |  |

Table S4. Parameter estimates and HPD CI for all parameters of the contemporaneous (${Beliefs}_{tj}$→${Rumination}_{tj}$→${Depression}_{tj}$) Dynamic Structural Equation Model with positive metacognitive beliefs as the predictor.

| Par | Est. | 95% HPD CI | PSR | Ess |
| --- | --- | --- | --- | --- |
| a | 0.362 | [0.317, 0.406] | 1.001 | 11352 |
| b | 0.514 | [0.475, 0.553] | 1.000 | 10486 |
| $c^{'}$ | 0.097 | [0.066, 0.128] | 1.000 | 10370 |
| $\beta_{X}$ | 0.201 | [0.158, 0.243] | 1.000 | 7428 |
| $\beta_{M}$ | 0.130 | [0.094, 0.165] | 1.000 | 8918 |
| $\beta_{Y}$ | 0.066 | [0.041, 0.092] | - | - |
| $l_{X}$ | -0.001 | [-0.028, 0.025] | 1.003 | 2294 |
| $l_{M}$ | -0.002 | [-0.028, 0.025] | 1.005 | 395 |
| $l_{Y}$ | 0.000 | [-0.023, 0.024] | 1.001 | 456 |
| $\omega_{X}$ | -0.102 | [-0.146, -0.060] | 1.001 | 3727 |
| $\omega_{M}$ | -0.333 | [-0.399, -0.267] | 1.002 | 370 |
| $\omega_{Y}$ | -0.592 | [-0.677, -0.506] | 1.000 | 5575 |
| ${var(\mu}_{1j})$ | 0.002 | [0.001, 0.005] |  |  |
| ${var(\mu}_{2j})$ | 0.002 | [0.001, 0.004] |  |  |
| ${var(\mu}_{3j})$ | 0.002 | [0.001, 0.004] |  |  |
| ${var(\mu}_{4j})$ | 0.051 | [0.037,0.070] |  |  |
| ${var(\mu}_{5j})$ | 0.032 | [0.022, 0.045] |  |  |
| ${var(\mu}_{6j})$ | 0.012 | [0.007, 0.019] |  |  |
| ${var(\mu}_{7j})$ | 0.057 | [0.042, 0.079] |  |  |
| ${var(\mu}_{8j})$ | 0.040 | [0.029, 0.056] |  |  |
| ${var(\mu}_{9j})$ | 0.023 | [0.015, 0.033] |  |  |
| ${var(\mu}_{10j})$ | 0.031 | [0.018, 0.049] |  |  |
| ${var(\mu}_{11j})$ | 0.122 | [0.088, 0.171] |  |  |
| ${var(\mu}_{12j})$ | 0.221 | [0.164, 0.301] |  |  |

Table S5. Parameter estimates and HPD CI for all parameters of the lagged (${Focusing}_{\left( t-1 \right)j}$→${Rumination}_{\left( t-1 \right)j}$→${Depression}_{tj}$) Dynamic Structural Equation Model with attentional focusing as the predictor.

| Par | Est. | 95% HPD CI | PSR | Ess |
| --- | --- | --- | --- | --- |
| a | -0.069 | [-0.118, -0.020] | 1.000 | 3289 |
| b | 0.073 | [0.033, 0.113] | 1.000 | 20707 |
| $c^{'}$ | 0.012 | [-0.016, 0.040] | 1.000 | 10138 |
| $\beta_{X}$ | 0.200 | [0.155, 0.244] | 1.000 | 4426 |
| $\beta_{M}$ | 0.175 | [0.132, 0.216] | 1.000 | 9002 |
| $\beta_{Y}$ | 0.142 | [0.102, 0.182] | - | - |
| $l_{X}$ | -0.002 | [-0.027, 0.024] | 1.000 | 936 |
| $l_{M}$ | -0.002 | [-0.029, 0.024] | 1.003 | 564 |
| $l_{Y}$ | -0.001 | [-0.024, 0.026] | 1.000 | 444 |
| $\omega_{X}$ | -0.109 | [-0.155, -0.063] | 1.013 | 557 |
| $\omega_{M}$ | -0.185 | [-0.235, -0.136] | 1.000 | 525 |
| $\omega_{Y}$ | -0.118 | [-0.158, -0.077] | 1.000 | 3204 |
| ${var(\mu}_{1j})$ | 0.002 | [0.001, 0.005] |  |  |
| ${var(\mu}_{2j})$ | 0.002 | [0.001, 0.005] |  |  |
| ${var(\mu}_{3j})$ | 0.002 | [0.001, 0.005] |  |  |
| ${var(\mu}_{4j})$ | 0.056 | [0.041, 0.077] |  |  |
| ${var(\mu}_{5j})$ | 0.050 | [0.036, 0.069] |  |  |
| ${var(\mu}_{6j})$ | 0.028 | [0.018, 0.044] |  |  |
| ${var(\mu}_{7j})$ | 0.071 | [0.053, 0.098] |  |  |
| ${var(\mu}_{8j})$ | 0.030 | [0.019, 0.046] |  |  |
| ${var(\mu}_{9j})$ | 0.012 | [0.007, 0.020] |  |  |
| ${var(\mu}_{10j})$ | 0.046 | [0.029, 0.069] |  |  |
| ${var(\mu}_{11j})$ | 0.052 | [0.033, 0.078] |  |  |
| ${var(\mu}_{12j})$ | 0.032 | [0.019, 0.050] |  |  |

Table S6. Parameter estimates and HPD CI for all parameters of the lagged (${Shifting}_{\left( t-1 \right)j}$→${Rumination}_{\left( t-1 \right)j}$→${Depression}_{tj}$) Dynamic Structural Equation Model with attentional shifting as the predictor.

| Par | Est. | 95% HPD CI | PSR | Ess |
| --- | --- | --- | --- | --- |
| a | -0.077 | [-0.126, -0.027] | 1.000 | 3287 |
| b | 0.078 | [0.038, 0.118] | 1.000 | 22689 |
| $c^{'}$ | -0.002 | [-0.028, 0.024] | 1.000 | 10841 |
| $\beta_{X}$ | 0.190 | [0.146, 0.231] | 1.001 | 4321 |
| $\beta_{M}$ | 0.181 | [0.138, 0.223] | 1.000 | 8606 |
| $\beta_{Y}$ | 0.143 | [0.103, 0.182] | - | - |
| $l_{X}$ | -0.001 | [-0.026, 0.027] | 1.000 | 1211 |
| $l_{M}$ | -0.002 | [-0.028, 0.026] | 1.002 | 434 |
| $l_{Y}$ | 0.000 | [-0.024, 0.026] | 1.002 | 467 |
| $\omega_{X}$ | -0.093 | [-0.134, -0.056] | 1.003 | 431 |
| $\omega_{M}$ | -0.189 | [-0.237, -0.140] | 1.001 | 343 |
| $\omega_{Y}$ | -0.115 | [-0.157, -0.072] | 1.000 | 3295 |
| ${var(\mu}_{1j})$ | 0.002 | [0.001, 0.005] |  |  |
| ${var(\mu}_{2j})$ | 0.002 | [0.001, 0.005] |  |  |
| ${var(\mu}_{3j})$ | 0.002 | [0.001, 0.005] |  |  |
| ${var(\mu}_{4j})$ | 0.049 | [0.035, 0.069] |  |  |
| ${var(\mu}_{5j})$ | 0.051 | [0.037, 0.071] |  |  |
| ${var(\mu}_{6j})$ | 0.028 | [0.018, 0.045] |  |  |
| ${var(\mu}_{7j})$ | 0.072 | [0.053, 0.098] |  |  |
| ${var(\mu}_{8j})$ | 0.032 | [0.021, 0.049] |  |  |
| ${var(\mu}_{9j})$ | 0.009 | [0.005, 0.016] |  |  |
| ${var(\mu}_{10j})$ | 0.027 | [0.016, 0.043] |  |  |
| ${var(\mu}_{11j})$ | 0.051 | [0.034, 0.076] |  |  |
| ${var(\mu}_{12j})$ | 0.030 | [0.018, 0.046] |  |  |

Table S7. Parameter estimates and HPD CI for all parameters of the lagged (${Beliefs}_{\left( t-1 \right)j}$→${Rumination}_{\left( t-1 \right)j}$→${Depression}_{tj}$) Dynamic Structural Equation Model with positive metacognitive beliefs as the predictor.

| Par | Est. | 95% HPD CI | PSR | Ess |
| --- | --- | --- | --- | --- |
| a | 0.360 | [0.316, 0.403] | 1.002 | 2378 |
| b | 0.080 | [0.040, 0.119] | 1.000 | 10172 |
| $c^{'}$ | 0.009 | [-0.024, 0.042] | 1.000 | 8586 |
| $\beta_{X}$ | 0.200 | [0.156, 0.243] | 0.999 | 6403 |
| $\beta_{M}$ | 0.131 | [0.095, 0.166] | 1.000 | 6880 |
| $\beta_{Y}$ | 0.146 | [0.106, 0.186] | - | - |
| $l_{X}$ | -0.001 | [-0.028, 0.023] | 1.002 | 1997 |
| $l_{M}$ | -0.002 | [-0.028, 0.024] | 1.000 | 474 |
| $l_{Y}$ | 0.001 | [-0.024, 0.025] | 1.005 | 522 |
| $\omega_{X}$ | -0.101 | [-0.146, -0.061] | 1.018 | 457 |
| $\omega_{M}$ | -0.334 | [-0.400, -0.268] | 1.001 | 355 |
| $\omega_{Y}$ | -0.121 | [-0.161, -0.081] | 1.001 | 2053 |
| ${var(\mu}_{1j})$ | 0.002 | [0.001, 0.005] |  |  |
| ${var(\mu}_{2j})$ | 0.002 | [0.001, 0.005] |  |  |
| ${var(\mu}_{3j})$ | 0.002 | [0.001, 0.005] |  |  |
| ${var(\mu}_{4j})$ | 0.051 | [0.037, 0.071] |  |  |
| ${var(\mu}_{5j})$ | 0.033 | [0.023, 0.046] |  |  |
| ${var(\mu}_{6j})$ | 0.032 | [0.020, 0.050] |  |  |
| ${var(\mu}_{7j})$ | 0.056 | [0.041, 0.077] |  |  |
| ${var(\mu}_{8j})$ | 0.025 | [0.015, 0.039] |  |  |
| ${var(\mu}_{9j})$ | 0.017 | [0.010, 0.028] |  |  |
| ${var(\mu}_{10j})$ | 0.030 | [0.017, 0.047] |  |  |
| ${var(\mu}_{11j})$ | 0.123 | [0.089, 0.172] |  |  |
| ${var(\mu}_{12j})$ | 0.030 | [0.019, 0.047] |  |  |

**Supplementary analysis of higher-order lagged effects model (from *t-2* to *t-1* to *t*)**

We fitted three higher-order lagged ($X_{\left( t-2 \right)j}$→$M_{\left( t-1 \right)j}$→$Y_{tj}$) models using Bayesian Markov chain Monte Carlo estimation, with attentional focusing, attentional shifting, and positive metacognitive beliefs as separate predictors. Results showed that all Potential Scale Reduction Factor (PSR) values were below 1.1, indicating successful convergence of the Markov chains.

In the higher-order lagged model with attentional focusing as the predictor, neither the path from attentional focusing to rumination (*a* = 0.02, 95% HPD CI [-0.001, 0.06]) nor the direct path to depressed mood ($c^{'}$ = 0.01, 95% HPD CI [-0.02, 0.04]) was statistically significant, as their credible intervals included zero. Although rumination significantly predicted depressed mood (*b* = 0.08, 95% HPD CI [0.04, 0.12]), the average mediation effect was not significant ($E(a_{j}b_{j}$) = 0.002, 95% HPD CI [-0.001, 0.005]).

The model with attentional shifting as the predictor revealed a lagged effect on rumination (*a* = 0.02, 95% HPD CI [-0.01, 0.05]) and the direct path ($c^{'}$= 0.00, 95% HPD CI [-0.03, 0.03]) were not significant. Again, despite a significant *b* path (*b* = 0.08, 95% HPD CI [0.04, 0.12]), the mediation effect was non-significant (E ($a_{j}b_{j}$) = 0.002, 95% HPD CI [-0.001, 0.005]).

In contrast, the model with positive metacognitive beliefs as the predictor revealed a significant lagged effect on rumination (*a* = 0.04, 95% HPD CI [0.00, 0.07]). Coupled with the significant effect of rumination on depressed mood (*b* = 0.08, 95% HPD CI [0.04, 0.12]), the indirect mediation effect was significant (E ($a_{j}b_{j}$) = 0.003, 95% HPD CI [0.000, 0.007]), indicating that metacognitive beliefs exert a more enduring influence on depressed mood through rumination across time.

This dissociation highlights a key temporal difference: the effects of attentional control are more immediate and transient, primarily manifesting concurrently, while the effects of metacognitive beliefs are more robust and persist across longer time lags.

Table S8. Parameter estimates and HPD CI for all parameters of higher-order lagged (${Focusing}_{\left( t-2 \right)j}$→${Rumination}_{\left( t-1 \right)j}$→${Depression}_{tj}$) Dynamic Structural Equation Model with attentional focusing as the predictor.

| Par | Est. | 95% HPD CI |
| --- | --- | --- |
| a | 0.024 | [-0.009, 0.057] |
| b | 0.076 | [0.037, 0.115] |
| $c^{'}$ | 0.013 | [-0.016, 0.042] |
| $\beta_{X}$ | 0.200 | [0.154, 0.244] |
| $\beta_{M}$ | 0.186 | [0.142, 0.228] |
| $\beta_{Y}$ | 0.142 | [0.104, 0.181] |
| $l_{X}$ | -0.002 | [-0.028, 0.024] |
| $l_{M}$ | -0.004 | [-0.030, 0.025] |
| $l_{Y}$ | -0.002 | [-0.027, 0.026] |
| $\omega_{X}$ | -0.113 | [-0.159, -0.068] |
| $\omega_{M}$ | -0.136 | [-0.182, -0.089] |
| $\omega_{Y}$ | -0.124 | [-0.164, -0.083] |
| ${var(\mu}_{1j})$ | 0.002 | [0.001, 0.005] |
| ${var(\mu}_{2j})$ | 0.002 | [0.001, 0.005] |
| ${var(\mu}_{3j})$ | 0.002 | [0.001, 0.005] |
| ${var(\mu}_{4j})$ | 0.057 | [0.041, 0.078] |
| ${var(\mu}_{5j})$ | 0.051 | [0.037, 0.071] |
| ${var(\mu}_{6j})$ | 0.028 | [0.001, 0.005] |
| ${var(\mu}_{7j})$ | 0.023 | [0.015, 0.035] |
| ${var(\mu}_{8j})$ | 0.030 | [0.019, 0.046] |
| ${var(\mu}_{9j})$ | 0.015 | [0.009, 0.024] |
| ${var(\mu}_{10j})$ | 0.046 | [0.029, 0.069] |
| ${var(\mu}_{11j})$ | 0.046 | [0.029, 0.070] |
| ${var(\mu}_{12j})$ | 0.030 | [0.019, 0.047] |

Table S9. Parameter estimates and HPD CI for all parameters of higher-order lagged (${Shifting}_{\left( t-2 \right)j}$→${Rumination}_{\left( t-1 \right)j}$→${Depression}_{tj}$) Dynamic Structural Equation Model with attentional shifting as the predictor.

| Par | Est. | 95% HPD CI |
| --- | --- | --- |
| a | 0.023 | [-0.007, 0.053] |
| b | 0.081 | [0.041, 0.120] |
| $c^{'}$ | 0.001 | [-0.026, 0.029] |
| $\beta_{X}$ | 0.190 | [0.147, 0.232] |
| $\beta_{M}$ | 0.193 | [0.148, 0.236] |
| $\beta_{Y}$ | 0.142 | [0.103, 0.181] |
| $l_{X}$ | -0.001 | [-0.027, 0.024] |
| $l_{M}$ | -0.003 | [-0.032, 0.024] |
| $l_{Y}$ | 0.000 | [-0.025, 0.024] |
| $\omega_{X}$ | -0.095 | [-0.135, -0.057] |
| $\omega_{M}$ | -0.130 | [-0.174, -0.083] |
| $\omega_{Y}$ | -0.120 | [-0.162, -0.076] |
| ${var(\mu}_{1j})$ | 0.002 | [0.001, 0.005] |
| ${var(\mu}_{2j})$ | 0.002 | [0.001, 0.005] |
| ${var(\mu}_{3j})$ | 0.002 | [0.001, 0.005] |
| ${var(\mu}_{4j})$ | 0.049 | [0.035, 0.069] |
| ${var(\mu}_{5j})$ | 0.053 | [0.038, 0.073] |
| ${var(\mu}_{6j})$ | 0.029 | [0.017, 0.044] |
| ${var(\mu}_{7j})$ | 0.017 | [0.010, 0.026] |
| ${var(\mu}_{8j})$ | 0.031 | [0.020, 0.048] |
| ${var(\mu}_{9j})$ | 0.012 | [0.007, 0.019] |
| ${var(\mu}_{10j})$ | 0.025 | [0.015, 0.041] |
| ${var(\mu}_{11j})$ | 0.044 | [0.029, 0.065] |
| ${var(\mu}_{12j})$ | 0.030 | [0.018, 0.048] |

Table S10. Parameter estimates and HPD CI for all parameters of higher-order lagged (${Beliefs}_{\left( t-2 \right)j}$→${Rumination}_{\left( t-1 \right)j}$→${Depression}_{tj}$) Dynamic Structural Equation Model with positive metacognitive beliefs as the predictor.

| Par | Est. | 95% HPD CI |
| --- | --- | --- |
| a | 0.037 | [0.002, 0.071] |
| b | 0.077 | [0.037, 0.116] |
| $c^{'}$ | 0.010 | [-0.022, 0.043] |
| $\beta_{X}$ | 0.202 | [0.157, 0.244] |
| $\beta_{M}$ | 0.179 | [0.134, 0.223] |
| $\beta_{Y}$ | 0.144 | [0.104, 0.183] |
| $l_{X}$ | -0.002 | [-0.028, 0.023] |
| $l_{M}$ | -0.003 | [-0.029, 0.025] |
| $l_{Y}$ | 0.000 | [-0.026, 0.025] |
| $\omega_{X}$ | -0.105 | [-0.149, -0.062] |
| $\omega_{M}$ | -0.132 | [-0.179, -0.086] |
| $\omega_{Y}$ | -0.122 | [-0.163, -0.081] |
| ${var(\mu}_{1j})$ | 0.002 | [0.001, 0.005] |
| ${var(\mu}_{2j})$ | 0.002 | [0.001, 0.005] |
| ${var(\mu}_{3j})$ | 0.002 | [0.001, 0.005] |
| ${var(\mu}_{4j})$ | 0.052 | [0.037, 0.072] |
| ${var(\mu}_{5j})$ | 0.052 | [0.037, 0.074] |
| ${var(\mu}_{6j})$ | 0.030 | [0.019, 0.046] |
| ${var(\mu}_{7j})$ | 0.025 | [0.016, 0.038] |
| ${var(\mu}_{8j})$ | 0.027 | [0.017, 0.043] |
| ${var(\mu}_{9j})$ | 0.019 | [0.012, 0.030] |
| ${var(\mu}_{10j})$ | 0.031 | [0.018, 0.050] |
| ${var(\mu}_{11j})$ | 0.043 | [0.027, 0.065] |
| ${var(\mu}_{12j})$ | 0.031 | [0.019, 0.050] |

**Supplementary references**

Derryberry, D., & Reed, M. A. (2002). Anxiety-related attentional biases and their regulation by attentional control. Journal of Abnormal Psychology, 111(2), 225–236. https://doi.org/10.1037/0021-843X.111.2.225

Han, X., & Yang, H. (2009). Chinese Version of Nolen-Hoeksema Ruminative Responses Scale (RRS) used in 912 college students: Reliability and validity. Chinese Journal of Clinical Psychology, 17(5), 550–551.

He, S. (2021). Attentional Control Scale Factor Structure and Empirical Validity [Dissertation, Hunan Normal University]. https://doi.org/10.27137/d.cnki.ghusu.2020.002106

Kroenke, K., Spitzer, R. L., & Williams, J. B. (2001). The PHQ-9: Validity of a brief depression severity measure. Journal of General Internal Medicine, 16(9), 606–613. https://doi.org/10.1046/j.1525-1497.2001.016009606.x

McNeish, D., & MacKinnon, D. P. (2022). Intensive longitudinal mediation in Mplus. Psychological Methods, 30(2), 393–415. https://doi.org/10.1037/met0000536

Müller, C. L., Rosenkranz, T., Schiller, A., Takano, K., Watkins, E. R., Mühlberger, A., Funk, J., & Ehring, T. (2025). Repetitive negative thinking in daily life predicts psychopathology: Further validation of an ecological momentary assessment paradigm. Behaviour Research and Therapy, 193, 104830. https://doi.org/10.1016/j.brat.2025.104830

Nolen-Hoeksema, S., & Morrow, J. (1991). A prospective study of depression and posttraumatic stress symptoms after a natural disaster: The 1989 Loma Prieta earthquake. Journal of Personality and Social Psychology, 61(1), 115–121. https://doi.org/10.1037/0022-3514.61.1.115

Papageorgiou, C., & Wells, A. (2001). Positive beliefs about depressive rumination: Development and preliminary validation of a self-report scale. Behavior Therapy, 32(1), 13–26. https://doi.org/10.1016/S0005-7894(01)80041-1

Wang, W., Bian, Q., Zhao, Y., Li, X., Wang, W., Du, J., Zhang, G., Zhou, Q., & Zhao, M. (2014). Reliability and validity of the Chinese version of the Patient Health Questionnaire (PHQ-9) in the general population. General Hospital Psychiatry, 36 5, 539–544. https://doi.org/10.1016/j.genhosppsych.2014.05.021

Zhou, H., Liu, H., Ma, X., & Deng, Y. (2023). The psychometric properties of positive and negative beliefs about the rumination scale in Chinese undergraduates. BMC Psychology, 11(1), 107. https://doi.org/10.1186/s40359-023-01111-8
